# Examining the absorption of post-internship medical officers into the public sector at county-level in devolved Kenya: a qualitative case study

**DOI:** 10.1186/s12913-023-09928-0

**Published:** 2023-08-19

**Authors:** Yingxi Zhao, Daniel Mbuthia, Joshua Munywoki, David Gathara, Catia Nicodemo, Jacinta Nzinga, Mike English

**Affiliations:** 1 Nuffield Department of Medicine, NDM Centre for Global Health Research, University of Oxford, S Parks Rd, Oxford, OX1 3SY UK; 2 KEMRI-Wellcome Trust Research Programme, Nairobi, Kenya; 3 MARCH Centre, London School of Hygiene and Tropical Medicine, London, UK; 4 Nuffield Department of Primary Care Health Sciences, University of Oxford, Oxford, UK; 5 Department of Economics, Verona University, Verona, Italy

**Keywords:** Human resources for health, Labour market, Recruitment

## Abstract

**Background:**

After Kenya’s decentralization and constitutional changes in 2013, 47 devolved county governments are responsible for workforce planning and recruitment including for doctors/medical officers (MO). Data from the Ministry of Health suggested that less than half of these MOs are being absorbed by the public sector between 2015 and 2018. We aimed to examine how post-internship MOs are absorbed into the public sector at the county-level, as part of a broader project focusing on Kenya’s human resources for health.

**Methods:**

We employed a qualitative case study design informed by a simplified health labour market framework. Data included interviews with 30 MOs who finished their internship after 2018, 10 consultants who have supervised MOs, and 51 county/sub-county-level managers who are involved in MOs’ planning and recruitment. A thematic analysis approach was used to examine recruitment processes, outcomes as well as perceived demand and supply.

**Results:**

We found that Kenya has a large mismatch between supply and demand for MOs. An increasing number of medical schools are offering training in medicine while the demand for MOs in the county-level public sector has not been increasing at the same pace due to fiscal resource constraints and preference for other workforce cadres. The local Department of Health put in requests and participate in interviews but do not lead the recruitment process and respondents suggested that it can be subject to political interference and corruption. The imbalance of supply and demand is leading to unemployment, underemployment and migration of post-internship MOs with further impacts on MOs’ wages and contract conditions, especially in the private sector.

**Conclusion:**

The mismatched supply and demand of MO accompanied by problematic recruitment processes led to many MOs not being absorbed by the public sector and subsequent unemployment and underemployment. Although Kenya has ambitious workforce norms, it may need to take a more pragmatic approach and initiate constructive policy dialogue with stakeholders spanning the education, public and private health sectors to better align MO training, recruitment and management.

## Introduction

The estimated density of medical doctors in Kenya, 0.23 per 1000 population in 2020, is significantly lower than average for LMICs (1.4 per 1000 in 2017) [1], and there is disparity between counties in Kenya as well [2]. MO training in Kenya is now offered through 11 medical schools’ Bachelor of Medicine and Bachelor of Surgery (MBChB) programs [3]. This is followed by a one-year mandatory internship in one of 74 training hospitals across the country [4] before they are officially licensed and registered. In public sector facilities, the staffing norms and standards guideline published by the national Ministry of Health recommends 2 MOs at level 3 (health centre), 16 at level 4 (primary hospital) and 50 at level 5 (secondary hospital) in the health system [5]. Salary cost for MOs during their internship year is covered by the national government, whereas post-internship MO salaries are paid by county governments in a public sector facility after government devolution [6], recommended monthly salaries for MOs and other cadres are provided in Table 1 [7, 8].

**Table 1:** Salary and renumeration costs of different cadres in Kenya

Cadre	Salary costs in the public sector	Other benefits
**Medical officer interns**	Ksh 145,000 / USD 1,200 per month	Extraneous allowance, housing allowances, risk allowances (roughly Ksh 40,000, ~ USD 330) and specifically for MOs also emergency call allowances (Ksh 30,000, ~ USD 250)
**Medical officer**	Ksh 250,000 / USD 2,100 per month	
**Registered clinical officer**	Ksh 100,000 / USD 840 per month	
**Enrolled/registered nurse**	Ksh 94,000 to 100,000 / USD 790 to 840 per month	

Source [7, 8]

The expansion of medical training in Kenya, with public universities doubling or even tripling their intakes and some government-sponsored students in private universities, was aimed at helping the country achieve high quality universal health coverage (Table 2). In the period between 2005 and 2009 ninety-five percent (1678/1772) graduating medical doctors were recruited to deliver health care services in the public sector [4, 9]. Data for the period 2015 to 2018, after devolution and individual county governments became responsible for health care delivery including local health workforce planning and recruitment, suggest only 45% (825/1,800) newly qualified and registered MOs were employed in the public sector [10]. These data suggest low absorption of MOs is now a major contributor to shortages in the qualified medical workforce rather than production challenges.

**Table 2:** Major events related to MO training, recruitment and management

Year	Events and implications
**2010–2018**	**Expansion of medical schools** as only 2 medical school before 2010 and an increase to 11 in 2018, meanwhile number of annual outputs increased from 287 in 2006 to 628 in 2019
**2010–2013**	**New constitution in 2010, new government formed after general election** in 2013 and devolution of the healthcare system, leading to the national government became responsible for planning and regulation for health and medical education and establishing norms and standards, while 47 devolved county governments are responsible for workforce planning and recruitment
**2013**	**Collective bargaining agreement (CBA)** drafted by the doctors’ union and the Ministry of Health on 300% pay-rise for doctors, review of job groups, recruitment, deployment and promotions of doctors; though county governments were not signatories to the agreement
**2014**	**Ministry of Health published Human Resources for Health Norms and Standards Guidelines for the Health Sector,** which required each Level 3 health centre to have at least two MOs and each Level 4 primary hospital to have 17

Source: [2, 4, 5, 10–13]

In this study, we aimed to examine how post-internship MOs are recruited to the public sector at the county-level in Kenya. We drew on interview data with different stakeholders to understand the recruitment processes as well as perceived demand and supply for MOs.

## Materials and methods

### Study design and framework

We employed a qualitative case study design as this allowed for in-depth exploration of the complex processes of county-level health workforce planning. We defined “the absorption of post-internship medical officers into the public sector” as our phenomena and a generic Kenya “county-level” as our unit of analysis. We drew upon three sources of qualitative data: interviews with junior MOs who finished their internship after 2018, consultants who have supervised MOs, and county and sub-county-level managers who are involved in the planning and recruitment of MOs.

As we sought to understand how and why (not) post-internship MOs are recruited into the public sector, our study was informed by a simplified version of health labour market framework, which primarily focuses on the supply and demand for workforce [14, 15]. Workforce supply is often driven by both the labour and education sector, and it includes the number and availability of trained workers and also the workers’ willingness to accept pay and conditions for certain jobs [14]. The demand for workforce is largely determined by what the government (and other sectors) are willing to pay to hire healthcare workers, while this is usually associated with need which is derived from population and disease burden, demand and need are two different concepts [14, 16].

### Data collection

The data presented in this paper were collected as part of two wider studies aimed at (a) understanding the internship experience of medical officers in Kenya and (b) examining the county-level human resources for health (HRH) management capacity in Kenya. Semi-structured interviews with 30 junior medical officers and 10 consultants were conducted as part of the internship experience study between June and Sept 2021, which focus on interns’ experience including of applying for and securing jobs. Individual or group interviews with 51 county-level and subcounty-level managers were conducted as part of the HRH management capacity study in two selected Kenyan counties between Sept 2021 and March 2022, focusing on recruitment processes for MOs and other cadres (Table 3). In each case respondents were recruited using a snowballing approach or a purposive approach until data saturation was reached, with consideration of respondent characteristics (internship hospital, current occupation, affiliation etc.) to achieve variation and gain a broader perspective of the phenomenon. Regular meetings among the interviewers were set up during data collection to monitor progress and discuss preliminary findings for quality assurance.

**Table 3:** Data sources and collection

Respondents	Sampling	Respondent characteristics	Data collection approach	Interview focus
**30 junior medical officers who finished internship within 3 years**	Snowballing approach through facilitated introductions or referral by an interviewee; individuals were selected also considering different internship hospitals and current occupation	⦁ Undergraduate training: 29 respondents trained in public universities, 1 trained in private universities ⦁ Internship hospital type: 25 interned in public hospitals, 3 in mission hospitals, 1 in private hospital, and 1 in military hospital ⦁ Internship hospital level: 14 interned in level 5 hospitals and 16 in level 4 hospitals ⦁ Current occupation: 26 currently work as medical officers, 2 work as researchers, 1 work as resident, and 1 work in business	⦁ Semi-structured interviews ⦁ In-person or online in English between June and Sept 2021	⦁ Wellbeing, educational and work environment during the internship, ⦁ Experiences of applying and securing post-internship jobs
**10 consultants with experience supervising interns**	Snowballing approach through facilitated introductions or referral by an interviewee; individuals were selected considering different specialty	⦁ Internship hospital type: 8 respondents work in public hospitals, 1 work in mission hospital, 1 work in private hospital ⦁ Internship hospital level: 3 work in level 6 hospitals, 4 work in level 5 hospitals, 3 in level 4 hospitals ⦁ Specialty: 2 work in surgery, 2 work in internal medicine, 5 work in paediatric, 1 work in obstetrics and gynaecology	⦁ Semi-structured interviews ⦁ In-person or online in English between June and Sept 2021	⦁ Observations of interns’ experiences during internship and employment
**51 county-level and subcounty-level managers**	Two counties were purposively selected based on their HRH density and geographic locations; individuals were selected purposefully covering different departments and affiliations	⦁ County: 14 respondents come from county A, 37 from county B ⦁ Affiliation: 11 respondents work as county health department officials, 2 work as county public service board officials, 2 work as county other department officials, 17 work as subcounty health department officials, 19 work as public facility managers	⦁ Semi-structured individual or group interviews (22 individual interviews, 8 group interviews involving 29 participants) ⦁ In-person in English between Sept 2021 and March 2022	⦁ Respondents’ role as HRH managers ⦁ Counties’ HRH interventions ⦁ How medical officers, clinical officers and nurses are recruited and deployed in counties

### Data analysis

Semi-structured interviews were audiotaped and transcribed, with all personally identifiable information removed and replaced by unique anonymous codes, and imported into Nvivo to manage coding and analysis. While data collection was conducted sequentially, at the data analysis phase we combined all interview data together, trying to examine and contrast different stakeholders’ perspectives on the recruitment processes as well as perceived demand and supply for MOs.

We used a thematic analysis approach to code all data. While there are different versions of the labour market framework with varied level of details, our analysis was guided by a simplified framework which focuses on the demand vs. supply model [14, 15]. The first author read through all transcripts, carried out some initial deductive and inductive coding according to the elements of the original framework, and core thematic areas and sub-themes were further refined and summarized as we familiarized ourselves with the data. For example, recruitment process was added as a theme despite not being an element of the labour market framework. Preliminary findings were presented to the other two data collectors as well as all other authors who were not directly involved in coding to triangulate and increase the trustworthiness of the findings.

### Reflexivity

This study is a collaboration between Kenyan and international researchers. The first author (YZ) is a Chinese researcher with a master’s in public health and past research experiences on human resources for health and health systems, but limited past experiences working in Kenya. Two other co-authors (DM and JM) are two Kenyan researchers with public health training and research experiences in Kenya’s health systems. YZ, DM and JM are not medical doctors/officers and at the time of data collection all affiliated with a Kenyan research organization. These three researchers co-led the interview guide development and the interview processes. Researchers’ backgrounds and affiliations were shared with participants prior to the interview. After each interview, researchers debriefed and discussed their perspectives and also how previous experiences and interactions with the participants may influence their interpretation, to promote continued reflexivity. While the analysis for the study was performed by YZ himself, DM, JM and other co-authors who are researchers from Kenya or have lived and worked in Kenya, also reviewed his findings to ensure that they were a true reflection of the data to minimize potential bias.

## Results

**Fig. 1:**
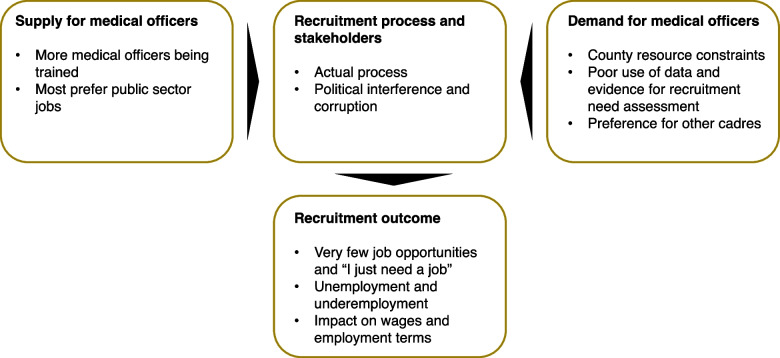
Summary of identified themes and subthemes

In this section, we summarized the major themes and subthemes emerged from the analysis. Figure 1 presents these themes and subthemes which are identified and guided by the simplified labour market framework. Besides issues related to supply and demand, we also summarized how the recruitment is actually done (process and stakeholders) and the impact on labour market (outcome).

### Recruitment process and stakeholders

Recruitment for MOs and other healthcare professionals starts with the county Department of Health identifying gaps and sending in requests to the county secretary and the human resources committee, which will review budgets and approve/reject such requests. The recruitment process is carried out by the county Public Service Board, and their duties include advertisement, conducting interviews alongside technical experts from the Department of Health, and recruitment. Prior to devolution, recruitment was done by the national government with direct posting of MOs to health facilities. According to our respondents, health facility managers are not directly involved in the MO recruitment process at the county-level, therefore are rarely able to retain interns except for a few locum positions if the hospitals themselves are able to pay for these positions.

*“It's a bit complex, because we really do not know what happens, sometimes you just bump into an advert in the media and then after that you will find new people being posted to the hospital. You will not know when the interviews were conducted, who conducted interviews and under what terms these people have come, you don't know, you’ll just be told these people have come to work here.” (Consultant, C10 – L4 Public – Internal medicine)*

Political interference and corruption affect the recruitment process for cadres including MOs, according to our respondents including MOs and some managers from sub-counties. For instance, MOs would be employed despite not attending interviews while some are asked for a bribe to get jobs. Some MOs mentioned that only people from the local county and community are likely to be hired. However, county government managers suggested that local people are more likely to be retained in the long-term, which is a consideration during recruitment.

*“I'm sorry to say this. You find that, you know, employment is a political tool. If, if today I am the governor, I want to reward my, my people. Most likely you, you reward them through, through employment.” (County B, B20)*

*“Another thing is that they employ based from tribe. If, like somebody like me coming from Western Kenya and I wanted to be employed around this area [County B] or something is very difficult.” (Medical officer, M10 – L5 Public – Private MO)*

### Supply of medical officers

Both MO respondents and county-level managers commented that there are many more MOs being trained in Kenya currently, as there are an increasing number of medical universities including several private universities now providing MBChB training, and several public universities increased their annual intakes. Additionally, people are also seeking medical training abroad in neighbouring countries including Uganda and Tanzania as well as Russia.

*“Medical officers are finding it harder because first of all almost every other university is offering medicine nowadays. So the number of medical officers out here is quite high. So if the supply is high, and the demand is constant, the demand by the medical facilities is constant, then that means that means that the people will not be employed as much as they used to be previously.” (Medical officer, M23 – L4 Public – Public MO)*

Out of these graduates, the supply for public sector, i.e. post-internship MOs that are willing to accept pay and conditions for public sector jobs is high. While MOs have different preferences for their future practice locations due to the attributes of different sectors, according to our respondents the majority prefer public sector jobs due to better job security and remuneration, “lower workloads” and better supervision. Thus, the public sector is felt to be “easier to work in” while being more respected and improving the possibility of being sponsored for graduate studies. In comparison, a few MOs prefer private sector jobs especially in a few prestigious private facilities because of better infrastructure and particular specialties, however the job security, working hours and remuneration are worse in most private facilities when compared with the public sector. Some other MOs also prefer faith-based facilities due to some particular specialty or their personal value.

*“We all wish as doctors especially myself to work where my job security is good. Working with a private facility in Kenya as a doctor, your job security is not guaranteed. You can wake up in the morning you go, you find- they fire employees. So it‘s not a place that I wish to be.” (Medical officer, M10 – L5 Public – Private MO)*

### Demand for medical officers in the public sector

Managers suggested that there is severe fiscal resource constraint in county governments to finance health workforce recruitment. MOs commented that health is not considered a priority by the county governments, which don’t want to further increase their salary expenditure. One county manager explained that the salaries of doctors and nurses are a bit high, and as salaries are included in recurrent expenditure that county governments are legally required to limit, it’s very challenging to further increase the budget for the workforce.

*“I think overriding reason is finances. We keep on hearing from our county and other county governments about the wage bill, wage bill...the main reason for not employing is not that they’re not there, they are there, they’re qualified they’re jobless, but there isn't enough money to employ to employ all, adequate numbers. That is the main reason.” (County B, B34)*

*“The issue of counties is finances that is one because unless they are given, what they received is a package, yeah. And unless the salaries are factored in for those doctors from the national government, the county cannot absorb. They will always squeeze if they can, maybe one has retired or one has left the service, that’s when they can absorb at least one or two. The vacancies are there, it's not that they are not there. They are there. But that package does not allow additional employment.” (County A, A05-08)*

Interestingly, interviewees from the two counties commented that recruitment for all cadres in the public sector including MOs is based on need, with gap analyses conducted by the relevant department and then forwarded to the county Public Service Board for consideration of recruitment. However, some sub-county respondents also commented that they think recruitment was not based on data or evidence, and that the county government especially the Public Service Board has poor understanding, planning and budgeting capacity for the health workforce, with poor use of data and evidence for forecasting and recruitment.

*“When you want to employ a new medical officer, we first do a needs assessment for our county, whether we require just a plain medical officer or specialised medical officers. and then from there we give our views that we require, this is the number we require whether it is medical officers, specialised officers. And then I think the rest, when we give our review as a committee, now the rest is from there up to the County Public Service Board” (County B, B01-05)*

*“I agree with you but what I am feeling is that the Public Service Board should actually be capacity built to understand what is Human Resources in Health and who is supposed to be employed? …if we are crying that we have a shortage and the Public Service Board does not understand what we need then they need to be upgraded and understand what is happening.” (County B, B13-17)*

Across the health system, despite staffing norms and guidelines requiring two MOs to be stationed at each Level 3 health centre, most of these facilities have never had an MO and are not considered suitable for such cadres because they lack the infrastructure and equipment that would warrant deploying better qualified staff. For example, these facilities do not have operating theatre and many commented that MOs is not largely needed when there is only outpatient services, which could be and already being done by clinical officers.

Difference in salary costs were also an important issue for county governments when considering demand for new MOs. According to our interviewees, county governments prefer recruiting other cheaper cadres including nurses, clinical officers and even social workers instead of MOs. Instead of recruiting MOs, county governments also benefit from the annual posting of MO interns paid by the national government to their major county hospitals that are internship training centres, these interns are treated as “free labour”—even though they are not fully registered to practice, and this can discourage counties to further recruitment MOs. County government respondents also commented that they prefer recruiting nurses and clinical officers as they can work in both higher-level hospitals and lower-level health centres and dispensaries. From our county and subcounty managers perspective, the recommended staffing norms are not always useful for actual employment planning.

*“I think it's cheaper to have clinical officers, let me start there. And they usually look for medical officers where, in the facilities where there are actual operating theatres because of C sections and all those procedures they think medical officers should do, and consultants. So if you have like or very large county without facilities that have an operating theater, they will tend to take on to clinical officers and nurses.” (Medical officer, M12 – L4 Public – Private MO)*

### Recruitment outcomes: employment, terms and wages

Some MO respondents commented that decentralisation, the collective bargaining agreement together with health worker strikes led to way fewer job opportunities for MOs becoming available both in the public sector and private/mission sectors. They contrasted this with the increasing outputs from training schools and most MOs reported that they “just wanted a job anywhere (they) could find”. While some county-level respondents commented that the county has employed more staff overall since devolution, many MOs remain jobless and unemployed in Kenya. MOs commented that a several months’ gap between graduation and starting their first job has become the norm. According to our respondents, many MOs are now employed on very short-term contracts instead of in permanent and pensionable positions by the county government. In these short-term positions they are underemployed e.g. working very few hours, and forced to locum and engage in dual-practice. This led to MOs being demotivated, and some respondents have considered exiting the clinical workforce or migrating to other countries.

*“But many people in fact, I have very many colleagues right now who are at home without work…So you find someone having, in fact, most of the interns, post internship, many people have like one year to two years job gaps, you can imagine, they have never taken back to hospital, not because they don't want, there are no opportunities.” (Medical officer, M01 – L5 Public - Research)*

*“And again you see when you employee somebody on contract then the next time you are not renewing the contract, how do you want to manage that person? Even you personally, how do you arrange your life? You are on contract, you are earning today, then tomorrow you are not earning, you are looking for a job elsewhere.” (County B, B01-05)*

The imbalance in supply and demand also had an impact on wages and employment terms especially in the private sector. While the private sector provides some employment opportunities, participants highlighted issues around underpayment, recruiting being done on a short-term or locum basis and the expectation of longer working hours compared to the public sector. For many MOs, the private sector was less attractive and only seen as a “stepping stone” to better employment opportunities.

*“And if you apply for a job somewhere. You find these private facilities that will offer you say half of what you are getting [during] your internship, and they are already qualified doctors with a registration number...” (Medical officer, M03 – L4 Mission – Public MO)*

*“Most of the private hospitals are giving doctors, you just work per working hours and it's not guaranteed. This is depending on the patient flow. So you find this month you might be called for locum, then suddenly next month you're being told, ‘okay, it’s Covid. There are very few patients, so you're going to be on a pay cut’.” (Medical officer, M01 – L5 Public - Research)*

## Discussion

In summary, we found that Kenya has a large and probably growing mismatch between supply and demand for MOs. There are an increasing number of medical schools producing higher numbers of MO graduates, these doubled from 287 in 2006 to 628 in 2019 [2, 4, 10]. In comparison, despite an increase in positions in the public sector with more MOs being employed (from 1715 in 2008 to 3712 in 2018 [10, 17]), the demand for MOs in the public sector has not been keeping same pace with the supply due to financial constraints and preference for other (cheaper) cadres according to our interviewee. As supply of MOs is largely driven by the education sector which is separate from the health sector, if medical education is not well-regulated this could compromise training quality as seen in other countries [18] but also could neglect the market need and demand with a drift to universities operating on a “training for profit” basis. The imbalance between supply and demand would therefore lead to resource waste considering that medical education is also subsidised by government. As for recruitment process, where MO recruitment occurs, the process is led by the county Public Service Board and may be subject to political interference and corruption [19]. The resulting surplus of MOs has led to unemployment, underemployment and migration of post-internship MOs, all this seems to be impacting MOs’ wages, reducing these in private sector.

Macroeconomically restrictions on public sector wage bills limit the counties’ ability to further absorb MOs into the public sector [20, 21]. While the Public Finance Management Act (2012) and subsequent 2015 supplement governing county budgets did not state the ceiling for HRH costs directly, they did require county governments to spend less than 70% of their total budget on recurrent costs, and personnel emoluments should not exceed 35% of county revenue [22], however this is often violated in practice [23]. Specifically for the health sector which takes up roughly one third of a county’s total budget, personnel costs contributed to around 60% of a county’s health budget in 2020 and in some counties as high as 78% [24], especially those who inherited the provincial hospitals with a larger staff establishment. Correspondingly, Kenya has had several rounds of public sector hiring freezes, therefore it would be challenging for counties themselves to further increase their human resources number and raise expenditure for staffing.

Counties have used different mechanisms to avoid these costs such as opting for contract-based staff instead of permanent and pensionable terms [21] and used project-based hiring such as the Universal Health Coverage programme and Covid-19 emergency responses to employ additional short-term staff [25], similar to many other countries [26]. With the expansion of medical schools and more MO interns, counties also rely on these interns who are posted annually and paid by the national government and consider them as “substitutes” for MOs despite that they are not legally fully qualified. Similarly, in some counties specialist resident trainees fill the gap in providing service delivery, which both threaten quality of service delivery but also discourage potential demand for MO recruitment. As a result, despite the number of MOs and specialists in the public sector increasing from 1715 in 2008 to 3712 in 2018, jobs have not been increasing at the same rate as the supply which itself is still not adequate. The stock of MOs estimated in 2020 is 12,792 [2] whereas the national HRH norms and standards developed in 2014 requires 19,255 MOs [5]. Despite the shortage, only 825 medical graduates from 2015 to 2018 have been absorbed by the public sector, which is less than half of the total MO graduates for these years [10].

Despite recruitment being said to be preceded by a needs assessment, respondents commented that the national guideline for staffing norms and standards is not helpful as the target is high, unrealistic and hard to implement. While the national staffing norms and standards require each Level 3 health centre to have at least two MOs and each Level 4 primary hospital to have 17, currently there are on average 0.23 MOs and 5.95 MOs available nationwide at those levels suggesting an enormous gap [27]. The lack of basic infrastructure and equipment such as theatres in those facilities made county managers believe that they do not need MOs but prefer to recruit other cadres to staff these facilities. More practical staffing norms and planning tools would be needed to assist county governments in projecting their workforce number and skill-mix required. Additionally better enforcement of these guidelines potentially linking with financial measures could help compliance in counties.

While the public sector is not able to fully absorb post-internship MOs, the private sector has also not taken up those available and even started to exploit MOs given the excess supply of labour. Salaries and remunerations in the private sector are not well-regulated and inferior than in the public sector and employment terms are shorter contracts with poor job security. Such unfair treatment is also seen in other settings of decentralisation and privatisation as national civil services protections no longer apply and health workers are more exposed to unfair hiring, disciplinary and work practices [28]. The faith-based sector, despite making important contributions to the health system in Kenya, only contributes to 11% of all facilities [29] and is unlikely to absorb large numbers of qualified MOs. Nonetheless we have not specifically explored recruitment processes for these sectors in this work.

The interviews with MOs and subcounty managers also suggested that they considered decentralisation led to political interference and corruption during the recruitment process, not only for MOs but for all cadres. As powers have been decentralised to the local government and not necessarily the local health departments, local elites could use recruitment as a tool to consolidate political support, enlarge voter base [12, 30] and reinforce tribalism and clientelism as seen in other countries [31]. Health facilities currently have very limited participation in recruitment and deployment other than a few that could afford locum positions, and facility-in-charges voiced concerns over workforce coordination as they are not involved in recruitment processes. This is in line with previous findings that show county hospitals now lack autonomy and control over human resources management which negatively impacts service delivery [32]. The recruitment process needs to be further optimised to minimise political interference, corruption and also ensure all relevant stakeholders are included in the process.

These findings have clear policy implications for Kenyan policy makers. There needs to be constructive policy dialogue between different stakeholders in different sectors to improve workforce planning, recruitment and management especially matching the quantity of medical school intake and MO production with the health needs and economic capacity so as to maintain a market equilibrium, alongside broader workforce planning for specialists, nurses, clinical officers and other cadres [2]. Such multi-ministerial and stakeholder discussions should include stakeholders from the supply side with Ministry of Education and all medical schools to reconsider medical students’ intake, and the demand side with MOH, county health departments, Kenya Health Professions Oversight Authority together with the broader national and county governments including Ministry of Finance and public service boards. There is a need to expand fiscal space for more health workforce expenditures and prepare achievable and routine workforce and costing plans. Facilities and labour unions should also be included in those dialogues to discuss options with employment terms and arrangements, and regulatory councils should also be involved to monitor and enforce county governments to employ more MOs if county hospitals are to train and receive MO interns and resident trainees.

Other options such as further recentralising HRH management functions to the national level could potentially address some challenges with poor planning and management capacity, nepotism and corruption with recruitment processes and maldistribution of the workforce between counties. Countries like Tanzania partially re-centralised their recruitment systems for health workers, teachers and accountants in 2006, 24 years after decentralisation, to better coordinate planning, training and recruitment of the workforce [33]. Similar structures for centralised management also exist in Uganda’s “Health Service Commission” and Kenya’s “Judicial Service Commission” and “Teachers Service Commission”, and currently the discussion of a potential “Kenya National Health Service Commission” is ongoing with the proposed function of training, recruiting, deploying, transferring and disciplining the health workforce [34]. However, it should be also acknowledged that re-centralisation of HRH management is not without challenges, there could be maldistribution and absenteeism in rural areas, as seen pre-devolution in Kenya [35, 36].

Several limitations should be considered when interpreting our findings. To start with, we were only able to interview managers from two counties and respective sub-counties, due to fieldwork arrangements. While the counties were selected based on workforce density and geographic locations, we acknowledge that there could be variations in recruitment practices in each county, and these findings might not be generalisable to all counties e.g. Nairobi City County. Second, we used a mix of convenience, purposeful and snowball sampling approaches to include respondents from different sectors and roles, however we have a rather limited number of respondents from the county public service board and no respondent from county finance departments. Further investigation with these organisations and others such as the county secretary could help us better understand how to expand counties’ fiscal space for the health workforce. Third, we specifically focused on the public sector as the majority of hospital services are still provided through this sector especially to the poor and those in rural areas. We were unable to interview stakeholders relevant to private and faith-based sectors recruitment even though we acknowledge their role in service delivery and as important employers for MOs. Forth, we also acknowledge that some of our statements regarding the number of medical graduates, or public sector employment have different time points as stated in the introduction section. While these are all the most recent and publicly available data, they might be directly comparable. Last, while we did not focus on Covid-19, it should be noted that this study was conducted during the pandemic where they would had been a higher workforce demand, though it is also likely the pandemic and lockdown might have led to financial constraints of the governments.

## Conclusion

We examined how post-internship MOs are absorbed into the public sector at the county-level in Kenya by understanding MOs’ supply, demand, recruitment processes and decision-making, and outcomes. These findings highlight the imbalance between MO supply due to significantly increasing medical student training and only a slowly increasing demand for MO in the public sector due to resource constraints and preference for other healthcare cadres. The mismatched supply and demand led to substantial MO unemployment and underemployment in the public sector risking them being unfairly treated by the private sector. While ambitious health workforce norms indicate major deficits in the medical workforce, fiscal constraints threaten the ability to close these gaps.

## Data Availability

All data relevant to the study are included in the article.
