# Correction: Examining the absorption of post-internship medical officers into the public sector at county-level in devolved Kenya: a qualitative case study

**DOI:** 10.1186/s12913-023-09992-6

**Published:** 2023-09-05

**Authors:** Yingxi Zhao, Daniel Mbuthia, Joshua Munywoki, David Gathara, Catia Nicodemo, Jacinta Nzinga, Mike English

**Affiliations:** 1 Nuffield Department of Medicine, NDM Centre for Global Health Research, University of Oxford, S Parks Rd, Oxford, OX1 3SY UK; 2 KEMRI-Wellcome Trust Research Programme, Nairobi, Kenya; 3 London School of Hygiene and Tropical Medicine, MARCH Centre, London, UK; 4 Nuffield Department of Primary Care Health Sciences, University of Oxford, Oxford, UK; 5 Department of Economics, Verona University, Verona, Italy

**Correction to:** ***BMC Health Services Research*** **(2023) 23:875.**

10.1186/s12913-023-09928-0.

Following publication of the original article [1], the authors identified errors in the affiliation assignment, caused by a typesetting mistake: affiliation 3 (instead of affiliation 2) was incorrectly assigned to Mike English as a second affiliation.

This error is corrected in the affiliations list of this Correction article and the original article [1] has been updated.
